# Intravenous Iron (III) Hydroxide Polymaltose Complex Infusion Related Severe Prolonged Hypophosphatemia

**DOI:** 10.7759/cureus.79868

**Published:** 2025-03-01

**Authors:** Mohamed Bashir, Asma Aljaberi

**Affiliations:** 1 Internal Medicine, Tawam Hospital, Al Ain, ARE; 2 Endocrinology, Tawam Hospital, Al Ain, ARE; 3 Medicine, College of Medicine and Health Sciences, Al Ain, ARE

**Keywords:** fibroblast growth factor 23 (fgf23), intravenous iron infusion, iron deficiency anemia, iron (iii) hydroxide polymaltose complex (ipc), severe hypophosphatemia

## Abstract

Intravenous iron infusion-related hypophosphatemia is an increasingly recognized phenomenon, potentially underdiagnosed in clinical practice. This case report describes the first known instance to the best of our knowledge, of severe hypophosphatemia following iron (III) hydroxide polymaltose complex (IPC) infusion in a 60-year-old woman with multiple comorbidities. The patient, with a history of diabetes mellitus type 2, hyperlipidemia, stable stage 3 chronic kidney disease, iron deficiency anemia, and osteoporosis, was found to have severe asymptomatic hypophosphatemia (<0.1 mmol/dL) during a general workup. She exhibited mild chronic numbness of the hands and feet, restless leg syndrome, and muscular pain in her shoulders, though her clinical examination was unremarkable. The patient had received three IPC infusions over two months prior to presentation. She was admitted and treated with intravenous phosphate followed by oral sodium phosphate. The patient remained well and asymptomatic throughout the treatment.

## Introduction

Severe hypophosphatemia is a significant clinical concern due to its potential to cause muscle weakness, respiratory failure, cardiac dysfunction, and neurological disturbances. Although relatively rare, it is a potentially life-threatening condition that can arise in various medical contexts. Common etiologies include inadequate dietary phosphate intake, excessive renal excretion, or internal redistribution of phosphate. Among these, one increasingly recognized but underdiagnosed cause is intravenous iron infusion [1].

Intravenous iron formulations are widely used to treat iron deficiency anemia, particularly in patients who are intolerant to oral iron supplements or require rapid replenishment of iron stores. While the therapeutic benefits of intravenous iron are well-established, its adverse effects on phosphate metabolism are less well-understood. Recent studies have identified a significant association between specific intravenous iron formulations, particularly ferric carboxymaltose (FCM), and the development of hypophosphatemia. The pathogenesis of this condition is primarily attributed to an increase in fibroblast growth factor 23 (FGF23), which reduces renal phosphate reabsorption and decreases the synthesis of 1,25-dihydroxy vitamin D, leading to a hypophosphatemic state [2,3].

Although FCM is most frequently implicated in such cases, other iron formulations have generally been considered to have a safer profile. Iron (III) hydroxide polymaltose complex (IPC) is one such formulation, known for its favorable safety profile and tolerability [4-6]. To date, IPC has not been commonly associated with severe hypophosphatemia, making this case particularly noteworthy.

## Case presentation

Patient history and presentation

A 60-year-old postmenopausal woman with chronic kidney disease stage 3, hyperlipidemia, controlled hypertension, type 2 diabetes mellitus, vitamin B12 deficiency, thalassemia trait, iron deficiency anemia, and osteoporosis (DXA scan showed T-score -3 at spine), with slightly elevated vitamin D on a supplement, and no history of fracture, presented for routine follow-up. Regular blood tests revealed severe hypophosphatemia at <0.1 mmol/L (reference range: 0.80-1.45 mmol/dL). 

She reported mild numbness in her hands and feet, and restless leg syndrome, consistent with her chronic iron deficiency anemia, as the patient used to have these symptoms previously even when phosphate level was normal. She also gave a history of chronic shoulder pain. Physical examination was unremarkable with normal cardiopulmonary, musculoskeletal, and neurological exams.

Chart review revealed that the patient received metformin-sitagliptin (1000-50 mg PO BID), empagliflozin (25 mg PO OD), perindopril (5 mg PO OD), iron-folic acid 1 cap (PO OD), alendronate 70 mg (PO Q 7 days), calcium carbonate-cholecalciferol (600 mg-200 units PO OD), magnesium oxide (500 mg PO OD), and rosuvastatin (20 mg PO HS). For the management of iron deficiency anemia, she received three intravenous IPC infusions over the two months prior to presentation.

Treatment and outcomes

**Figure 1:**
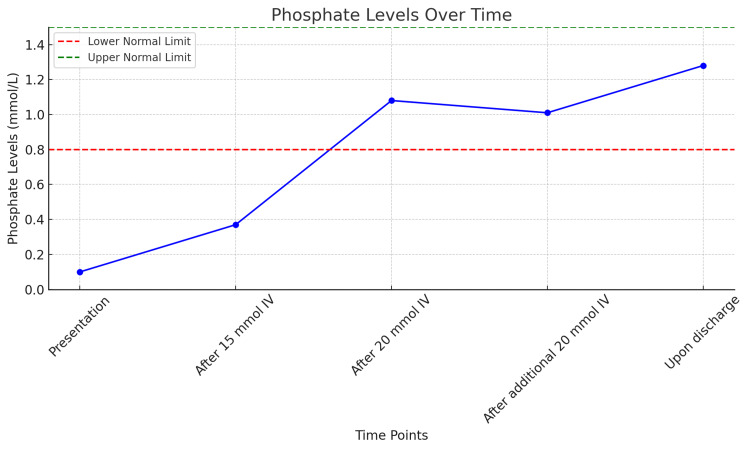
Phosphate levels over time.

The patient was advised for admission for correction of the severe deficiency and further work-up. She was admitted to the hospital with continuous telemetry cardiac rhythm monitoring. She received a total of 55 mmol of intravenous sodium phosphate, administered in three divided doses, along with oral sodium phosphate (500 mg twice daily). Her phosphate levels improved to 1.28 mmol/L (Figure 1); other electrolytes were within normal limits.

Additional investigations showed no iron deficiency, normal parathyroid hormone, high vitamin D (175 nmol/L), normal ferritin level, and stable kidney function. Urine analysis showed glycosuria and proteinuria (Table 1). 

**Table 1:** Laboratory values. MCV: mean corpuscular volume; MCH: mean corpuscular hemoglobin; MCHC: mean corpuscular hemoglobin concentration: RDW: red blood cell distribution width; MPV: mean platelet volume.

Test name	Value	Reference range
Sodium	134 mmol/L	135-145 mmol/L
Potassium	4.6 mmol/L	3.5-5.0 mmol/L
Chloride	99 mmol/L	98-107 mmol/L
Bicarbonate	22 mmol/L	22-28 mmol/L
Creatinine	106 μmol/L	60-110 μmol/L
Urea	7.10 mmol/L	2.5-6.4 mmol/L
Calcium	2.43 mmol/L	2.15-2.55 mmol/L
Calcium (corrected)	2.44 mmol/L	2.15-2.55 mmol/L
Magnesium	0.69 mmol/L	0.70-1.00 mmol/L
Phosphate (presentation)	<0.1 mmol/L	0.80-1.50 mmol/L
Phosphate (15 mmol IV)	0.37 mmol/L	0.80-1.50 mmol/L
Phosphate (20 mmol IV)	1.08 mmol/L	0.80-1.50 mmol/L
Phosphate (additional 20 mmol IV)	1.01 mmol/L	0.80-1.50 mmol/L
Phosphate (discharge)	1.28 mmol/L	0.80-1.50 mmol/L
eGFR	49 ml/min	>60 ml/min
Urinary creatinine	5.51 mmol/L	2.47-19.2 mmol/L
Urinary phosphate	<0.10 mmol/L	13-42 mmol/L
Parathyroid hormone	2.1 pmol/L	1.6-6.9 pmol/L
Alkaline phosphatase	39 IU/L	35-104 IU/L
Albumin	39 g/L	35-52 g/L
HbA1c	6.3%	<5.7%
Vitamin B12	334 pmol/L	128-648 pmol/L
Iron	12.0 μmol/L	5.8-35.8 μmol/L
Ferritin	277 μg/L	30-400 μg/L
Kappa light chain	39.30 mg/L	3.30-19.90 mg/L
Lambda light chain	40.80 mg/L	5.71-26.30 mg/L
K/L ratio	0.96	0.26-1.65
WBC	5.9 x 10^9^/L	4.0-11.0 x 10^9^/L
RBC	4.77 x 10^12^/L	4.50-5.50 x 10^12^/L
Hemoglobin	108 g/L	120-160 g/L
Hematocrit	0.34 L/L	0.37-0.47 L/L
MCV	71.5 fL	81-01 fL
MCH	22.6 pg	27-32 pg
MCHC	317 g/L	320-360 g/L
Platelets	333 x 10^9^/L	150-450 x 10^9^/L
RDW	17.2	11.5-14.5
MPV	8.60 fL	7.4-10.4 fL
Urine Protein	1+	
Urine Glucose	3+	

The electrocardiogram showed no conduction abnormalities or arrhythmias and no significant changes compared to her previous recordings. She remained asymptomatic during admission and was discharged in a stable condition after two days.

## Discussion

Intravenous iron infusion-related hypophosphatemia has been an emerging entity globally. The exact incidence of iron infusion-induced hypophosphatemia is unknown but based on Bishay et al., the incidence ranges between 2.1-86% [7]. It is mostly transient and clinically insignificant. It is primarily linked to an increase in levels of fibroblast growth factor 23 (FGF23), which is a hormone encoded by the moGene *FGF23* and mainly synthesized by the bone matrix and bone marrow cells. While the underlying pathophysiology remains unclear, it is thought that *FGF23* plays a crucial role in regulating phosphate homeostasis. One of its key functions is to inhibit renal phosphate reabsorption through the proximal tubules of the kidneys, thereby contributing to the maintenance of phosphate balance. Additionally, *FGF23* exerts an inhibitory influence on the synthesis of 1,25-dihydroxy vitamin D, resulting in a reduction in enteral phosphate absorption. These combined effects act synergistically to decrease total body phosphate levels [1,3].

After reviewing different search engines, PubMed and Google Scholar, we found that this is the first reported case of IPC-induced severe asymptomatic hypophosphatemia after a short period of time, which raises intriguing considerations regarding the safety profile of IPC compared to other intravenous iron formulations. There is a case series of two reported cases of moderate to severe IPC-induced hypophosphatemia after using 1 gm per month for a period exceeding 12 months [7]. Historically, IPC has been lauded for its favorable tolerability and minimal adverse effects [4,5], contrasting with the more commonly implicated ferric carboxymaltose (FCM) in hypophosphatemia cases [1].

This case challenges conventional perceptions of IPC's safety, necessitating a critical reevaluation of its potential metabolic repercussions. Furthermore, the pathophysiological mechanisms underlying IPC-induced hypophosphatemia remain elusive, particularly given the rarity of prior reported cases. Understanding these mechanisms is imperative for elucidating the risk factors predisposing individuals to this complication and implementing targeted preventive strategies. Moreover, heightened vigilance and systematic monitoring of phosphate levels post-IPC infusion are warranted to promptly detect and manage hypophosphatemia, particularly in patients with predisposing comorbidities.

## Conclusions

This case report highlights a rare but significant adverse effect of IPC infusion, presenting as severe hypophosphatemia in a patient with multiple comorbidities. Despite IPC’s established safety profile, this case emphasizes the need for increased vigilance and routine monitoring of serum phosphate levels following IPC administration, particularly in patients with existing risk factors. The findings challenge the conventional perception of IPC’s safety and call for further research to elucidate the underlying mechanisms of IPC-induced hypophosphatemia. Understanding these mechanisms is crucial for developing preventive and management strategies. Clinicians should be aware of this potential complication and ensure timely intervention to mitigate adverse outcomes in affected patients.
